# High-throughput SSR marker development and its application in a centipedegrass (*Eremochloa ophiuroides* (Munro) Hack.) genetic diversity analysis

**DOI:** 10.1371/journal.pone.0202605

**Published:** 2018-08-22

**Authors:** Jianjian Li, Hailin Guo, Yi Wang, Junqin Zong, Jingbo Chen, Dandan Li, Ling Li, Jingjing Wang, Jianxiu Liu

**Affiliations:** Institute of Botany, Jiangsu Province and Chinese Academy of Sciences, Nanjing Botanical Garden, Mem. Sun Yat-Sen, Nanjing, Jiangsu, China; National Cheng Kung University, TAIWAN

## Abstract

Centipedegrass (*Eremochloa ophiuroides* (Munro) Hack.) is a perennial, warm-season C4 grass species that shows great potential for use as a low-maintenance turfgrass species in tropical and subtropical regions. However, limited genetic and genomic information is available for this species, which has impeded systematic studies on the enhancement of its turf quality and resistance against biotic and abiotic stress. In this study, Illumina HiSeq high-throughput sequencing technology was performed to generate centipedegrass transcriptome sequences. A total of 352,513 assembled sequences were used to search for simple sequence repeat (SSR) loci, and 64,470 SSR loci were detected in 47,638 SSR containing sequences. The tri-nucleotides were the most frequent repeat motif, followed by di-nucleotides, tetra-nucleotides hexnucleotides, and pentanucleotides. A total of 48,061 primer pairs were successfully designed in the flanking sequences of the SSRs, and 100 sets of primers were randomly selected for the initial validation in four centipedegrass accessions. In total, 56 (56.0%) of the 100 primer pairs tested successfully amplified alleles from all four centipedegrass accessions, while 50 were identified as polymorphic markers and were then used to assess the level of genetic diversity among 43 centipedegrass core collections. The genetic diversity analysis exhibited that the number of alleles (Na) per locus ranged from 3 to 13, and the observed heterozygosity (Ho) ranged from 0.17 to 0.83. The polymorphism information content (PIC) value of the markers ranged from 0.15 to 0.78, and the genetic distances (coefficient Nei72) between the accessions varied from 0.07 to 0.48. The UPGMA-based dendrogram clustered all 43 core collections into two main groups and six subgroups, which further validated the effectiveness of these newly developed SSR markers. Hence, these newly developed SSR markers will be valuable and potentially useful for future genetic and genomic studies of *E*. *ophiuroides*.

## Introduction

Simple sequence repeats (SSRs) are a valuable source of genetic markers because of their abundance, high rate of polymorphism, ubiquitous distribution throughout the genome, codominant inheritance, high extent of allelic diversity, and ease of assay by PCR [1,2]. Thus, SSRs are considered excellent molecular markers in studies of germplasm characterization, genetic diversity, and genetic mapping [3,4]. However, the traditional development of SSR markers has relied on the screening of genomic libraries using repetitive probes and the sequencing of positive clones, which is time-consuming and requires the use of specialized laboratory equipment [5]. However, recent advances in next-generation sequencing (NGS) technologies provide a cost-effective, convenient and reliable approach for sequence information acquisition in non-model species and greatly accelerated the development process for molecular markers [6,7]. RNA-Seq, which is based on NGS, is a high-throughput technology that has great advantages in obtaining a large amount of sequence data for SSR mining [8]. Using transcriptome data resources, rapid progress in the development of SSR loci has been made in many green plant species [9]. Recently, a large amount of SSR markers were developed in forage and turfgrass crops such as perennial ryegrass [10], Italian ryegrass [11], alfalfa [12], hemarthria [13], red clover [14], orchardgrass [15], sudangrass [16], common bermudagrass [17], creeping bentgrass [18], seashore paspalum [19], and zoysiagrass [20]. To date, there is not a large quantity of SSR markers developed in centipedegrass, with the exception of a recent report on EST-SSR development from a cold-stressed transcriptome of centipedegrass [21].

Centipedegrass (*Eremochloa ophiuroides* (Munro) Hack.) is an important turfgrass that belongs to warm-season (C4) perennial grass species and is distributed extensively in South-East Asia and United States. *E*. *ophiuroides* is a native grass in South and Central China [22,23] and is known for its good adaptation to infertile soils and a range of climatic conditions [24,25]. *E*. *ophiuroides* has great potential as a low-maintenance turf and is often referred as ‘lazy man’s grass’ or ‘poor man’s grass’ because of its lower management requirements than those of most warm-season turfgrasses [26]. Centipedegrass usually presents a broad resistance to insect and disease infestations and shows excellent heat tolerance as well [26]. These outstanding characteristics make *E*. *ophiuroides* a popular turfgrass in tropical and subtropical regions. Moreover, centipedegrass is also used as a forage grass in Japan for its heavy grazing-tolerance [24].

Over the past two decades, a series of studies, including accession identification, an analysis of genetic diversity, and construction of genetic map in centipedegrass, was performed based on the use of limited universal molecular markers [27–31]. Some efforts in improving the lawn traits of centipedegrass, including traditional selection breeding [24,32,33], irradiation mutagenesis [34–36] and somatic variation, have also been carried out [25,37–39]. Despite progress in preliminary studies on genetic analysis and germplasm innovation, genetic and genomic information on this turfgrass species is still largely limited. Thus far, less than one hundred nucleotide sequences of DNA have been deposited in a public database (National Center for Biotechnology Information), which is markedly incomparable to that for other turfgrass plants, e.g., ryegrass, festuca, bermudagrass, or zoysiagrass. The paucity of available information on the nucleotide sequences has hindered its genetic and genomic studies, such as the large-scale development of molecular markers, the construction of high-density linkage maps, and gene discovery.

To further complement the genomic sequence information and the number of molecular markers, in this study we conducted large-scale SSR mining employing the RNA sequencing (RNA-Seq) data of centipedegrass leaf, stolon and spikes based on the high-throughput Illumina HiSeq 2000 platform. The resultant SSR sequences were characterized and validated through the successful amplification of randomly selected target loci across a selection of four distinct *E*. *ophiuroides* accessions. The newly developed SSR markers were subsequently utilized to assess the genetic diversity level of core collections, including 43 centipedegrass accessions from diverse geographic origins. The datasets and results reported in the present study provide a public resource and information for future genetic studies and breeding programs in *E*. *ophiuroides*.

## Materials and methods

### Plant materials and isolation of RNA and DNA

Two *E*. *ophiuroides* accessions E092 and E092-1 were used for cDNA sequencing. The accession E092 is a wild-type with red-purple stolons and spike tissues during its developmental stages, and it was originally collected from Chongqing city in the Southwest China. The accession E092-1 is a natural mutant with uniform green stolon and spike tissues and was isolated from E092. Initially, four *E*. *ophiuroides* accessions including two purple-stolon accessions (E092 and E022) and two green-stolon ones (E092-1 and E039) were used for validating the SSR primers, and then, 43 accessions from the *E*. *ophiuroides* core collection were adopted to test the SSR markers and assess the genetic diversity level (Table 1). All these accessions of *E*. *ophiuroides* were maintained by the Main Warm-season Turfgrass Germplasm Resource Preserving Centre, Nanjing Botanical Garden Men. Sun Yat-Sen, Jiangsu Province and Chinese Academy of Sciences, Nanjing, China. All the plant materials were grown in plastic pots (13 cm top diameter × 10 cm bottom diameter × 11 cm depth), with a mix of soil, sand and peat at a ratio of 1:1:1, and were cultivated in a greenhouse under natural sunlight, with an average temperature of 30°C day /20°C night and a relative humidity of ~80%.

**Table 1:** The germplasm collections of *E*. *ophiuroide* used in this study.

No.	Sample	Source
1	E-004	Fuzhou, Fujian, Eastern China
2	E-006	Putuoshan, Zhejiang, Eastern China
3	E-013	Yongtai, Fujian, Eastern China
4	E-015	Lushan, Jiangxi, Central China
5	E-017	Hangzhou, Zhejiang, Eastern China
6	E-019	Hangzhou, Zhejiang, Eastern China
7	E-022	Wuxi, Jiangsu, Eastern China
8	E-041	Lingchuan, Guangxi, South China
9	E-042	Taiping, Anhui, Eastern China
10	E-047	Lianyungang, Jiangsu, Eastern China
11	E-055	Guamngzhou, Guangdong, South China
12	E-061	Heyuan, Guangdong, South China
13	E-063	Yingde, Guangdong, South China
14	E-065	Yingde, Guangdong, South China
15	E-072	Guilin, Guangxi, South China
16	E-074	Yongzhou, Hunan, Central China
17	E-077	Zhangjiajie, Hunan, Central China
18	E-078	Yichang, Hubei, Central China
19	E-084	Anshun, Guizhou, Southwestern China
20	E-087	Guiyang, Guizhou, Southwestern China
21	E-091	Chongqing, Southwestern China
22	E-092-1	Chongqing, Southwestern China
23	E-097	Xinyang, Henan, Central China
24	E-098	Xinyang, Henan, Central China
25	E-099	Jinzhai, Anhui, Eastern China
26	E-102	USA
27	E-112	Xinjin, Sichuan, Southwestern China
28	E-115	Yiyang, Hunan, Central China
29	E-124	Changsha, Hunan, Central China
30	E-131	Yiyang, Hunan, Central China
31	E-134	Guiyang, Guizhou, Southwestern China
32	E-135	Guiyang, Guizhou, Southwestern China
33	E-141	Feixi, Anhui, Eastern China
34	E-144	Xinyang, Henan, Central China
35	E-145	Xinyang, Henan, Central China
36	E-152	Xinyang, Henan, Central China
37	E-154	Liuhe, Jiangsu, Eastern China
38	E-155	Lianyungang, Jiangsu, Eastern China
39	E-158	USA
40	E-182	Lanxi, Zhejiang, Eastern China
41	E-183	Nanjing, Jiangsu, Eastern China
42	E-187	Yixing, Jiangsu, Eastern china
43	E-188	Jinzhai, Anhui, Eastern China

The total RNA, which was required for the transcriptome sequencing, was extracted from the stolons, leaves and inflorescences of accessions E092 and E092-1 using the Trizol reagent (Invitrogen, Carlsbad, CA, USA) according to the manufacturer’s instructions. Prior to the RNA extraction, the tissue samples were frozen in liquid nitrogen and were homogenized by hand using a glass tissue grinder (DUALL 20, Kontes Glass Co.). The isolated RNA was treated with RNase-free DNase I (Ambion, Austin, TX, USA) to eliminate the potential genomic DNA. The RNA concentration and quality were determined by a NanoDrop 8000 spectrophotometer (NanoDrop, Wilmington, DE), and its integrity was confirmed using an Agilent 2100 Bioanalyzer (Agilent Technologies, Palo Alto, Calif.). Samples with an RNA Integrity Number (RIN) ≥ 7, and 28S:18S ratio ≥ 1.5:1, and total amount ≥ 3 μg were considered acceptable.

The total genomic DNA was isolated from the young leaves of each plant using the EZgene^TM^ SuperFast Plant Leaves DNA Kit (Biomiga, San Diego, CA, USA) following the manufacturer’s protocols for plant leaves with high levels of phenolic compounds and polysaccharides. The DNA was dissolved in 50 μL of sterile ultra-pure water, diluted to a final concentration of 30 ng/μL and stored at -20°C until the PCR analysis.

### cDNA library construction and Illumina sequencing

Illumina sequencing was performed at the Decode Genomics Ltd., in Nanjing, China, following the manufacturer’s protocols (Illumina, San Diego, CA). Briefly, the poly (A) mRNA was purified from the total RNA using Sera-mag Magnetic Oligo (dT) beads from Illumina, and then, the mRNA-enriched RNAs were chemically fragmented into short sequences using the fragmentation solution (Ambion, USA). The double-stranded cDNA was synthesized using the Superscript Double-Stranded cDNA Synthesis Kit (Invitrogen, USA). Finally, the pair-end RNA-seq libraries were constructed using the Illumina Paired End Sample Prep kit and were subsequently sequenced on the Illumina HiSeqTM 2000 platform [40].

### Data filtering and de novo assembly of cDNA

Prior to the transcriptome assembly, a stringent filtering criterion of an FPKM (fragments per kilobase of exon per million reads mapped) value of 1.0 in at least one sample or an FPKM value of 0.5 in at least two samples was used to filter the raw sequencing reads. The clean reads were obtained from the raw sequencing reads by removing the adapter sequences, the reads with more than 10% unknown nucleotides, and the low-quality reads (> 50% bases with quality value Q ≤ 5 in a read). The de novo assembly of the transcriptome was accomplished using all the clean reads and the Trinity program (version trinityrnaseq_r20140717) using the de Bruijn graph method and default settings [41].

### Sequence data

The assembled transcriptome sequences from the *E*. *ophiuroides* accessions E092 and E092-1 were deposited in the NCBI, with the BioProject accession PRJNA437781. They were *de novo* assembled from the raw sequencing data (7.5-fold coverage) of the accessions E092 and E092-1, which were deposited in the NCBI under the SRA accession number SRP134136 (SAMN08640788, SAMN08640789, SAMN08640790, SAMN08640791, SAMN08640792, SAMN08640793, SAMN08640794, SAMN08640795, and SAMN08640796).

### SSR motif detection and SSR marker development

We employed the software package Genome-wide Microsatellite Analyzing Tool Package (GMATA2.1) (http://sourceforge.net/projects/gmata/?source=navbar.) to mine the SSRs, perform the statistical analysis, and design primers from the identified SSR loci using constraints of more than five repeats and a motif length between 2 and 10 bp [42]. We followed the GMATA procedures described by Wang and Wang (2016) [42]. The primers were synthesized by Sangon Biotech Company (Shanghai, China).

Sequence IDs were given to the SSR-containing sequences used as templates for designing the primers (S1 Table). A marker name was assigned to each of the randomly selected SSR primer pairs for the PCR analysis. The marker name was comprised of the research center name “TJIB,” which stands for “Turfgrass Research Center of the Institute of Botany, Jiangsu Province and Chinese Academy of Sciences (JIB),” and the suffix showing the plant species *Eremochloa ophiuroides* (Eo) and the serial number.

### Genotyping the *E*. *ophiuroides* collections using the SSR markers

A subset of 100 SSR primer pairs was randomly selected for validating the SSR locus amplification by polymerase chain reactions (PCR). The primer pairs that produced a reproducible and clear amplicon of the expected size were then used for assessing the genetic diversity among the centipedegrass accessions. PCR amplification was conducted in a 10 μL reaction volume containing 5 μL of 2 x TaqPCR MasterMix (TsingKe Biological Technology Co., Beijing, China), 1 μL of primer pair (10 μM), 1.5 μL of genomic DNA (30 ng) and 2.5 μL of ddH_2_O. The PCR conditions comprised an initial denaturing step (95°C /3 min) followed by ten cycles of 94°C /30 s, 55–60°C /30 s, and 72°C /30 s, and then 25 cycles of 94°C /30 s, 55°C /30 s, and 72°C /30 s, and finally by an elongation step (72°C /10 min). The PCR products were separated on 8.0% non-denaturing polyacrylamide gels and were visualized by 0.1% silver nitrate staining. The band sizes were determined by comparing them against a DNA ladder.

### Data analysis

The genotyping data were used to determine the genetic relationships among the 43 *E*. *ophiuroides* core collections. The number of alleles (Na), the number of effective alleles (Ne), the observed heterozygosities (Ho), and the Shannon’s information index (I) were calculated using GenALEx software (version: 6.5) [43,44]. The polymorphic information content (PIC) value of the alleles revealed by each primer pair was calculated by the formula: *PIC* = 1−∑(*Pi*)^2^, where Pi is the frequency for the i^th^ microsatellite allele. The genetic distances across the collections were calculated with the POPGENE software (version 1.31; https://www.ualberta.ca/~fyeh/popgene_download.html) [45]. Based on Nei's unbiased measures of genetic distance, a cluster analysis of the 43 collections was carried out using the unweighted pair-group method with arithmetic average (UPGMA), and the dendrogram was subjected to 1000 bootstraps using the MEGA4 [46].

## Results

### Illumina paired-end sequencing and characterization of the sequencing reads

In this study, to remove the highly similar or redundant sequences, we merged sequences with a sequence identity higher than 95% using the CD-HIT-EST algorithm [47]. A total of 390,247,286 clean reads were generated using the Illumina Hiseq2000 system, and 352,513 assembled sequences were used for further analysis after adaptor removal. The length of the assembled sequences varied from 200 bp to > 16 kb, with the average of approximately 735 bp, and the CG content was approximately 48% (Table 2).

**Table 2:** Occurrence of microsatellites in *E*. *ophiuroides* transcriptome survey.

Category	Numbers
Total CleanReads	390,247,286
Maximum sequence length (bp)	16,303
Minimum sequence length (bp)	200
Average sequence length (bp)	734.79
GC content (%)	47.96
Total number of sequences examined	352,513
Total size of examined sequences (bp)	259,022,733
Total number of identified SSRs	64,470
Number of SSR containing sequences	47,638
Number of sequences containing more than one SSR	11,725

### SSR mining and characterization

The GMATA strategy was performed to search the SSR loci from the assembled transcriptome sequences. A total of 64,470 SSR loci were found in 47,638 SSR-containing transcriptome sequences. Of the SSR-containing transcriptome sequences, 11,725 contained more than one SSR locus (Table 2 and S1 Table).

**Fig 1:**
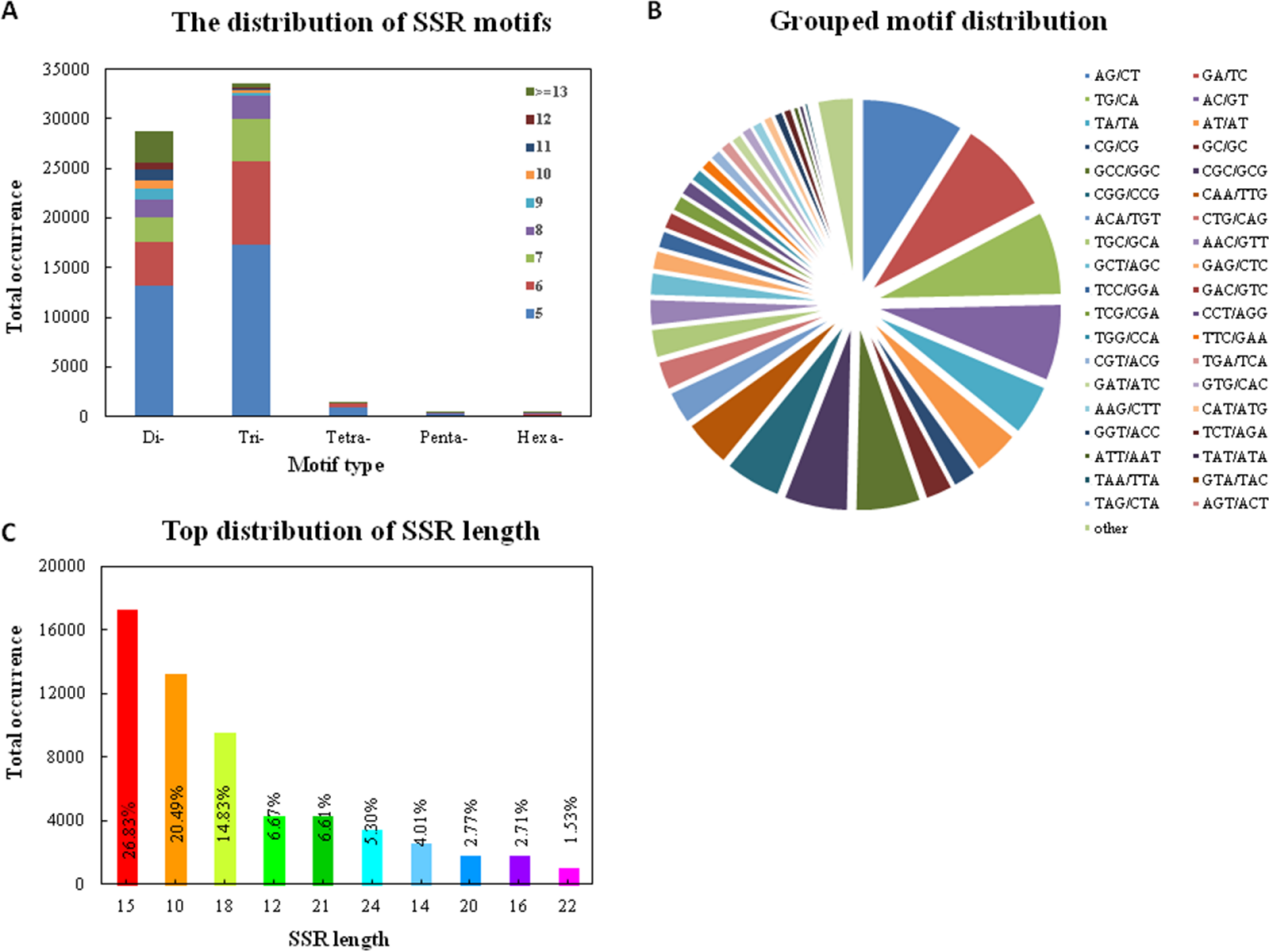
Statistical graphics of *E*. *ophiuroides* SSRs by the GMATA. (A) shows the distribution of SSR motifs, (B) shows grouped motif distribution, (C) shows Top distribution of SSR length.

Among all the SSRs detected, the most abundant repeat motifs were trinucleotides (33,614, 52.14%), followed by dinucleotides (28,783, 44.65%), tetranucleotides (1,399, 2.17%), hexnucleotides (331, 0.51%) and pentanucleotides (317, 0.49%) (Fig 1A, S2 Table). The highest frequency of the grouped SSR motif units was dimer AG/CT (5,774, 8.96%) followed by GA/TC (5,362, 8.32%), TG/CA (4,746, 7.36%), AC/TG (4,353, 6.75%), GCC/GGC (3,674, 5.70%) and CGC/GCG (3,613, 5.60%) (Fig 1B, S3 Table). The SSR length of 15 bp (17,296, 26.83%) was the most abundant, followed by an SSR length of 10 bp (13,212, 20.49) and then of 18 bp (9,563, 14.83) (Fig 1C, S4 Table).

### Development and validation of the SSR markers

A total of 48,061 non-redundant primer pairs were designed using Primer 3.0 software based on the criteria of the melting temperature, CG content, lack of secondary structure and length of amplification bands (S5 Table). The expected length of the target bands was between 100 bp and 400 bp.

Initially, a subset of 100 primer pairs was randomly selected for validating the SSR locus amplification. A total of 81 (81.0%) of the primer pairs tested successfully generated amplification products in at least one of the four *E*. *ophiuroides* accessions (E022, E039, E092, E092-1), and 56% of the primer pairs successfully amplified alleles from all four centipedegrass accessions. Of the 81 that amplified, 31 (38.27%) were monomorphic, and the other 50 (61.73%) were polymorphic between the four accessions and were selected for further evaluating the genetic diversity in the *E*. *ophiuroides* core collections (Table 3).

**Table 3:** Screening of SSR primer pairs by PCR in four *E*. *ophiuroides* accessions.

ID	Marker Name	SSR	Forward primer (5'-3')	Reverse primer (5'-3')	Product size (bp)	PCR amplification in			
						E092-1	E092	E022	E039
TR80_c0_g1_i1	TJIB.Eo_001	(CTC)12	AGTTCATTTCGCCAGCTCAT	TAAACTGCGTGCAGCAAAAC	195	**+**	**-**	**-**	**-**
TR993_c0_g1_i1	TJIB.Eo_002	(ACA)16	TGCATCAACGGAGAAAGACA	GGTTGCGATGATGAGTTGTG	141	**+**	**+**	**-**	**+**
TR1158_c0_g1_i1	TJIB.Eo_003	(GTT)11	CCACACCCTCGATTGATTCT	GGCGTTACAGGAGGTTAGCA	227	**+**	**-**	**-**	**+**
TR1654_c2_g1_i1	TJIB.Eo_004^※^	(CAA)13	GTGTGTGTGTGCTTGGTTGG	TGTGCCTCACAAATCGAGAC	197	**+**	**+**	**+**	**+**
TR1713_c3_g1_i1	TJIB.Eo_005	(TCT)10	ACCACAGGCAGGTGAGAGAC	ATGGCGTTGGTGTAGTCCAT	276	**+**	**+**	**+**	**+**
TR2338_c1_g1_i1	TJIB.Eo_006^※^	(GCC)5	GGTGGCGTTGTTTGCTATCT	CTGCTTCTTCTGCTTTCCGT	264	**+**	**+**	**+**	**+**
TR2644_c0_g1_i1	TJIB.Eo_007	(CTC)10	AATCTGGAACGCAACGAAAC	CTCTCCGTCCTCTCCTTTGA	275	**+**	**+**	**+**	**+**
TR2941_c0_g1_i2	TJIB.Eo_008^※^	(ACA)5	AGCAGGCGATAGACGAGTGT	CAGGTGGTGAGTCGTTGTTG	207	**+**	**+**	**+**	**+**
TR4044_c0_g1_i1	TJIB.Eo_009^※^	(GGT)10	CGTGATGAAAGCACCTGAGA	CTGGCTACCTTCCCTGCAC	274	**+**	**+**	**+**	**+**
TR5354_c0_g2_i1	TJIB.Eo_010^※^	(CTG)11	GGTGCTCAGTTGCAGCATAA	CGTCATACAACCGGAGGTG	183	**+**	**+**	**+**	**+**
TR5388_c0_g1_i2	TJIB.Eo_011	(ATA)11	TCGTCACACGGTGACAAAGT	AAGAGATCAGTGCTGGCCTA	152	**+**	**+**	**-**	**-**
TR5621_c0_g1_i1	TJIB.Eo_012	(TGC)5	CGAGTCATGGTCGCTGTAAA	AGCCAGAGTTCGAGCTTCAC	141	**-**	**-**	**-**	**-**
TR6729_c0_g1_i1	TJIB.Eo_013^※^	(ATA)11	ATAGCAGAAGCGAAGATGGC	AAAGTACTCCGTGGTCGTGG	173	**+**	**+**	**-**	**-**
TR7108_c0_g1_i1	TJIB.Eo_014^※^	(CAT)7cttg(CTC)5	GCCCAATCATCTCATCAACA	GATGAGGACGAAGAGGACGA	208	**-**	**+**	**+**	**+**
TR7160_c0_g2_i1	TJIB.Eo_015	(TGA)11	GGTCCCTTCTGGTGATTGTG	TTGCGCGTCGTTTATGATAG	227	**-**	**-**	**-**	**-**
TR7421_c0_g1_i1	TJIB.Eo_016^※^	(AGA)11	AGAGAAGAAAGGCCACACGA	GCTACCTGTTGCTGGCTCTC	107	**+**	**+**	**+**	**+**
TR8741_c0_g1_i1	TJIB.Eo_017^※^	(GAG)10	AGGGCTAGAATTAGGAGGCG	GCGTGAACGCTCACTCACT	107	**+**	**-**	**+**	**-**
TR9123_c0_g1_i1	TJIB.Eo_018	(GTC)5gtt(GTC)7	CGTTGTCGTCCTTGTTGTTG	CGAGCTAACCATCTAAGCGG	103	**-**	**-**	**+**	**-**
TR9124_c1_g1_i1	TJIB.Eo_019	(GT)8	TTCTTCATCGAGAACTGCCC	CGCCCATGAAAACCTACACT	148	**-**	**-**	**-**	**-**
TR9728_c0_g2_i1	TJIB.Eo_020^※^	(GCG)11	AGGGTACCATCCCCTTCCTT	TTCCCAACACCTGAATCACC	264	**+**	**+**	**+**	**-**
TR10202_c2_g1_i1	TJIB.Eo_021	(A)14	TTTGGGCTGCCGATATTAAG	CAGGGTGCTTCTCTCTCGTC	123	**+**	**+**	**+**	**+**
TR11518_c0_g1_i1	TJIB.Eo_022^※^	(CAA)11	AAACAATTGGCGGCAGTATC	TGTTTTGGTTTGTTCCAACG	132	**+**	**+**	**+**	**+**
TR12898_c0_g1_i1	TJIB.Eo_023^※^	(CAG)5	CGCAACAGATCAAGCAAGAG	GGCTGCTGATGATGATGATG	184	**+**	**+**	**-**	**+**
TR13811_c0_g1_i1	TJIB.Eo_024	(TCA)6	ATTTCTCTTGCAATGCCCTG	GGCAGCCTGTTTTTGATTGT	166	**+**	**+**	**+**	**+**
TR13845_c0_g1_i1	TJIB.Eo_025^※^	(A)12	ACTCCCAAACTGGACAATGC	AGCCATCTGCCCAACATAAT	118	**+**	**+**	**+**	**+**
TR13927_c0_g2_i1	TJIB.Eo_026	(ACG)5	CAAAGACCAGGTCGATGAGG	GTGTCTCGTGCGTGTTCTGT	279	**+**	**+**	**+**	**+**
TR14515_c0_g1_i1	TJIB.Eo_027	(TG)6	GGTGTCCGTTTATTTGTGGC	TGGCTGGCTCATAAGTGTTG	215	**-**	**-**	**-**	**-**
TR14777_c0_g1_i1	TJIB.Eo_028^※^	(CTG)10	ACGTTCTGATGGTGATGCTG	ACAGACTGGATGATGCACGA	236	**+**	**-**	**+**	**+**
TR15964_c0_g1_i1	TJIB.Eo_029^※^	(CAA)5	AACAACAGCAACAGCAGCAG	ATTTTCGACCGGGTTAGCTT	182	**+**	**+**	**+**	**+**
TR16265_c0_g1_i1	TJIB.Eo_030	(ATC)7	CGCATTATTGGTGAGGGAGT	CGACAAGTCAGGTTGCGATA	254	**-**	**-**	**-**	**-**
TR16281_c0_g1_i1	TJIB.Eo_031^※^	(CAC)13	GCGACGAGATGAGAATGAGA	CTAGGTGGGAGGATGCGAG	154	**+**	**+**	**+**	**+**
TR16284_c1_g1_i1	TJIB.Eo_032	(ACA)16	CAATTATCAGGCAGATGGGC	ATTTGGTGGTGAGCCGTTAG	109	**-**	**-**	**-**	**+**
TR16410_c1_g1_i1	TJIB.Eo_033	(GTG)12	ACCGTTATCTCTCGTGGTGG	CAATATCGCCAGACCGTTTT	155	**+**	**-**	**-**	**-**
TR17043_c1_g1_i1	TJIB.Eo_034	(TCA)10	CATCATGCTCATCATCGGAC	GGCTTCGCTTGAACTAGTCG	204	**-**	**-**	**-**	**-**
TR17216_c0_g1_i1	TJIB.Eo_035	(CAA)19	ATTGGCCATTGAGACCTCTG	TCGTTGAATTTGAATGCTCG	261	**+**	**+**	**+**	**+**
TR17372_c0_g1_i1	TJIB.Eo_036	(TTG)10	GATGTTGTTGGTGTTGGTGC	AAAACAATCGGCCATTCAAG	254	**+**	**+**	**+**	**+**
TR17509_c1_g1_i1	TJIB.Eo_037	(TGG)5	GCAACCGATTGGATTGAAGT	CACCAAACACGATTGTGGAC	185	**-**	**-**	**-**	**-**
TR18313_c0_g1_i1	TJIB.Eo_038^※^	(GCT)6gggctgcgactgatgcggcggcgg(CTT)5	ACCTGCTCAGGCTCAGCTC	ATAGCTCTCCAGCGACGATT	236	**+**	**+**	**+**	**+**
TR18539_c0_g2_i1	TJIB.Eo_039	(AAC)10	CCAGGAAAAAGAAGCAGCAG	TGTCAGTCTCTTGGTGGCTG	103	**-**	**-**	**-**	**-**
TR21460_c3_g4_i1	TJIB.Eo_040^※^	(CGT)5	CACCTGCTCAAGCACCATC	TCAAGGAACGAAACGAAACC	165	**+**	**+**	**+**	**+**
TR21890_c0_g1_i1	TJIB.Eo_041	(GCT)5	AGACGACTTCACGCGACTCT	GAGTTGCTGGAGGAAACAGC	192	**+**	**+**	**+**	**+**
TR23033_c0_g1_i1	TJIB.Eo_042	(GTT)10	CATGTTGTCGTCCTTGTGGT	ACGGCCGACTAACTAAAGCA	108	**-**	**-**	**-**	**-**
TR23780_c0_g1_i1	TJIB.Eo_043	(ACC)6aa(CAG)5(CAA)5	CGGAACTGCAAACCAAACTA	ACAATCAGACCTGTGGGCTT	141	**-**	**-**	**-**	**-**
TR23987_c0_g1_i1	TJIB.Eo_044^※^	(T)12	CTTTCAGAACGCGAGTTTCC	GGATACTATCAGCGCCCAAA	185	**-**	**+**	**+**	**+**
TR26689_c0_g1_i1	TJIB.Eo_045	(TCT)10	ACACTTGCTTCCCCAACATC	GGAAGTGACAGCAAGGAAGC	275	**+**	**+**	**+**	**+**
TR26876_c2_g2_i9	TJIB.Eo_046^※^	(A)11	AAAATCCATCCAACAGAGCG	ATGGGTCCTGTAAGCAGTGG	166	**-**	**+**	**+**	**+**
TR27357_c0_g3_i1	TJIB.Eo_047^※^	(ACA)10	GCAACATAATGGCTGCAATG	CAAGCGCCAATGCAATAGTA	237	**+**	**+**	**+**	**+**
TR27781_c0_g1_i1	TJIB.Eo_048	(CAG)7(CAA)5	TTACCAGTTCGTTGACGTGC	GTGCTGCTGCTGGATAGGAT	172	**+**	**+**	**+**	**+**
TR28391_c0_g1_i1	TJIB.Eo_049	(GT)6	ACATGAAGAGCTGCTGAGGC	CAAAACAAGTGCATGGCATC	110	**+**	**+**	**+**	**+**
TR28656_c0_g1_i1	TJIB.Eo_050	(TATG)11	GCAATTCAAGTTGTGGAGCA	TTTTGTAGGCCAACATTCTGC	170	**-**	**-**	**-**	**-**
TR29033_c0_g3_i1	TJIB.Eo_051^※^	(TTG)11	TGTAGTGCCGAGCAATTGAG	GCTGGCCAACCTGTAGAGAG	108	**+**	**+**	**+**	**+**
TR29155_c1_g3_i1	TJIB.Eo_052^※^	(CAA)5	GCAACCACAACACCAAACAC	GCAATCATGTCCGATTGATG	258	**+**	**+**	**+**	**+**
TR29597_c0_g1_i1	TJIB.Eo_053	(GTT)6	GTCGATGACACAAGCGACTG	TCCAGATTGAGTGCAACGAG	202	**+**	**+**	**+**	**+**
TR29883_c0_g1_i4	TJIB.Eo_054^※^	(GGA)10	CTTAGCCACCACCACATCCT	GTGACCTCTAGCCATCGGAG	161	**+**	**+**	**+**	**+**
TR30407_c0_g1_i1	TJIB.Eo_055^※^	(AG)12	GCTCGTGTGGACTACCAACA	TCCTCCTCTTCTCCTTGCTG	136	**+**	**+**	**+**	**+**
TR30845_c0_g1_i1	TJIB.Eo_056^※^	(ATG)10	ACCCACCAGACAGTGAGACC	CATTTTCCTTCCCTGACACG	202	**+**	**+**	**+**	**+**
TR31759_c0_g1_i1	TJIB.Eo_057	(ATC)11	TGGGTTGAAACAAGTTGACA	TGTTGCTGTTTGAAGTTTGATG	192	**+**	**+**	**+**	**+**
TR32331_c0_g1_i1	TJIB.Eo_058	(GAG)6	GAAAGAGGGGAGATGGACAA	ACTCACTCACTCCCCCACAC	191	**+**	**+**	**+**	**+**
TR33540_c0_g1_i1	TJIB.Eo_059	(ATG)10	TGTTCAAGTGCATGCAACAAT	ATTACTTGTGGGATGACGGC	244	**-**	**+**	**-**	**-**
TR33948_c0_g1_i1	TJIB.Eo_060^※^	(TGT)5(TGC)7	GCTCGTATTTGATTGGGCAT	GTCAATGTGCTCGAGTGTGC	250	**+**	**-**	**-**	**-**
TR34533_c0_g3_i1	TJIB.Eo_061^※^	(CTG)12	TCAGCAGTTGTGCTGGAATC	CCATGGGAGTGATGATGATG	276	**+**	**+**	**+**	**+**
TR34630_c0_g1_i3	TJIB.Eo_062^※^	(CCG)5	GAGATCTTCGCGGACATTG	ACATGACAGGACGACGCTC	237	**+**	**+**	**+**	**+**
TR35413_c0_g1_i1	TJIB.Eo_063^※^	(TG)8	GTGGCCGAAAAGAAGAAACA	TTTATCGAACGGAGTTTGCC	181	**+**	**+**	**+**	**+**
TR39034_c2_g2_i1	TJIB.Eo_064	(TGA)12	CTGTCGCTGTCACTGCCTAA	CGGACTTCTGTCAAAGGCAT	134	**-**	**-**	**-**	**-**
TR39482_c0_g1_i1	TJIB.Eo_065^※^	(CCT)7	TGAGAGAACCCTCATAACACAGA	GGAAAGGCTGTCTATGCTGC	142	**+**	**+**	**+**	**+**
TR39754_c0_g1_i1	TJIB.Eo_066^※^	(ATC)14	GCGCCTTCTCCTCTAACTCT	TTCTGTTGCGGAACTCCTCT	211	**+**	**+**	**+**	**+**
TR39866_c0_g1_i2	TJIB.Eo_067	(CAA)11	AGCGACAACGACTACGAGGT	AGGTACCGGATTGTGGGAGT	271	**+**	**+**	**+**	**+**
TR42732_c0_g1_i1	TJIB.Eo_068^※^	(GTT)12	ATGTGAACGCTTTCCTCTCG	AGACGATCAACGCAACAACA	230	**+**	**+**	**+**	**+**
TR43258_c0_g1_i1	TJIB.Eo_069	(CAA)10	ACCATGCCTCCAAATCCATA	AAGGTGCAGCTCATTGCTTT	268	**-**	**-**	**-**	**-**
TR44815_c0_g2_i1	TJIB.Eo_070	(AAT)13	ATCCCAATCAATACGGTCCA	TTTCTTTTTGCTACCGGACG	198	**-**	**-**	**-**	**-**
TR45627_c0_g1_i1	TJIB.Eo_071^※^	(GA)6	GGAGGAGGACCCAGAGAGAG	GAAATTGAGTAACGCACGCA	124	**+**	**+**	**+**	**+**
TR46377_c0_g1_i1	TJIB.Eo_072^※^	(GAA)10	CCTTCCCAAGACATTTTCCA	TAAGATCTTTTTGGGCTCGG	229	**+**	**+**	**+**	**-**
TR46548_c0_g1_i2	TJIB.Eo_073^※^	(CCG)11	TACAGCAATCCCTCCAGCTC	GAAACCATGGCCTCTTCCTC	278	**+**	**+**	**+**	**+**
TR46887_c0_g3_i1	TJIB.Eo_074^※^	(CTC)11	CTTTGAGAGGGCGCTTATTG	TGACCTTGAGTACGTGCTGG	115	**+**	**+**	**+**	**+**
TR46947_c0_g1_i1	TJIB.Eo_075^※^	(CTC)11	TCCTCCAAGACAATGCACG	CGTCTCCTCCTCTTCCTCCT	210	**+**	**+**	**+**	**+**
TR47176_c0_g1_i1	TJIB.Eo_076	(AAC)10	CCCTCGGACATCAATGGTT	GGTTGCTCGTTCTGGATGTC	179	**-**	**-**	**-**	**-**
TR47761_c0_g2_i1	TJIB.Eo_077^※^	(GTT)14	CCGTTATCTTGAGGCAGCTC	GGAAGACGAATGGCTTTCAG	135	**+**	**+**	**+**	**+**
TR48071_c1_g1_i2	TJIB.Eo_078^※^	(ACC)10	CATGAGCATCCACCTCCTCT	GTAAATGGTTTTGCAGCGGT	235	**+**	**+**	**+**	**+**
TR49631_c0_g1_i1	TJIB.Eo_079	(AAC)7	AACCTATTAGCAGTGTGTGCGA	TGTGCTCCTAAAACTGATTAGCTC	211	**-**	**-**	**-**	**-**
TR51210_c0_g2_i1	TJIB.Eo_080^※^	(ATC)14	AAGCGCCTTCTCCTCTAACC	ATTACTCGGAGGGTCCGTTT	195	**+**	**+**	**+**	**+**
TR51244_c7_g2_i1	TJIB.Eo_081	(CGC)12	TCGGTTTGAAAAATCCCAAC	TTCACTTTGCTTTGTGCCAG	259	**-**	**-**	**-**	**-**
TR51733_c0_g4_i1	TJIB.Eo_082^※^	(TTC)13	AGAGCAGTACTAGGCCGCAC	GCTCATCTCCATGGTTCGAT	156	**+**	**+**	**+**	**+**
TR51940_c0_g2_i1	TJIB.Eo_083	(CTT)15	TGTAAATCCGATGGTCTGGG	CCGTCGTCTTCCTCTACTGG	272	**-**	**+**	**+**	**-**
TR52704_c4_g2_i1	TJIB.Eo_084^※^	(CTC)10	GGGTACGTCATGATCGTGGT	CTCCGTCTCCTCCTCCTCTG	144	**+**	**+**	**+**	**+**
TR53512_c1_g1_i5	TJIB.Eo_085	(A)12	CACCTTCAAAACCTGCCATT	CACACACACCACTACGGGAG	258	**+**	**+**	**+**	**+**
TR53516_c0_g1_i1	TJIB.Eo_086^※^	(TGA)10	GGGCATAATGCTGTTGACAAT	AAGCATAGAACGAGCGCAAT	139	**-**	**+**	**+**	**+**
TR53584_c1_g1_i1	TJIB.Eo_087^※^	(ACA)6atcacgatcgc(CAA)6	TGTGCCATATTTTTCAAGCG	GGAATGCTCGAGAAACGAAG	255	**+**	**+**	**+**	**+**
TR53641_c0_g1_i1	TJIB.Eo_088	(GTT)10	GCCCCTATAAATGGAACCGT	CACAACCCAAATCATCACGA	144	**-**	**+**	**+**	**-**
TR53936_c0_g1_i1	TJIB.Eo_089^※^	(CAA)5	CCTCTTGCTCAGTTGCATCA	ACTACGAACAACCAGGTCGG	209	**+**	**+**	**+**	**+**
TR54047_c0_g1_i1	TJIB.Eo_090^※^	(CTAC)18	GGCCACACTGCTTTAACCAT	TCGCGGTAAATCTTTTCGAC	246	**+**	**+**	**+**	**+**
TR54725_c0_g4_i1	TJIB.Eo_091^※^	(TCA)5	CACACTTCCGAGGTGGACTT	AGGAGTGCCCAAATCACAAG	218	**+**	**+**	**+**	**+**
TR54851_c2_g5_i1	TJIB.Eo_092^※^	(TCC)10	CTGGCATCTCTTCTGGCAC	GAGGAGGAGGAGGAGGACAG	228	**+**	**+**	**+**	**+**
TR56883_c0_g2_i1	TJIB.Eo_093	(TTG)10	TACACTGGCTCAGCAAATCG	CAATGCAGTGTCGAAAGCAA	225	**+**	**-**	**+**	**-**
TR57592_c0_g1_i1	TJIB.Eo_094	(CAA)10	CAGAATGGGCCAGTTCAGTT	TCTGATCACCTGAATCGTCG	229	**-**	**-**	**-**	**-**
TR58461_c0_g2_i1	TJIB.Eo_095	(GAA)12	AACTCAGAACCGAAACGCTC	CTCGGAGACCTTATGGTGGA	213	**-**	**-**	**-**	**-**
TR59379_c0_g1_i2	TJIB.Eo_096	(CAT)5	GGTTGTGAATCATCAGCAGC	TAGCGGTGACAATGGATCAG	138	**-**	**-**	**+**	**+**
TR59407_c0_g1_i1	TJIB.Eo_097^※^	(CCT)10	CATAACAACGACACGATGGC	GAGGAGGAGGGAAAGAAGGA	264	**+**	**+**	**+**	**+**
TR60466_c0_g1_i1	TJIB.Eo_098	(CAG)6	TACGCGTTCAGCAATGCTAC	TTGCTGATTGTGGATGTGGT	108	**+**	**+**	**+**	**+**
TR60586_c0_g1_i1	TJIB.Eo_099	(TGC)5	TGACCACCTGTTGTTTGAGC	CCCAGAAGACGATGAACCAG	247	**-**	**-**	**+**	**-**
TR61247_c0_g4_i1	TJIB.Eo_100	(GTT)17	ACGATGAGTCTCGGTGGTTT	ATTGAGTCGTGCTTCGGACT	238	**+**	**-**	**-**	**-**

※ These marker loci showed polymorphic amplifications in the four accessions of *E*.*ophiuroides*.

### Evaluation of the genetic diversity within the *E*. *ophiuroides* core collections

The fifty polymorphic SSR markers were further used to assess the genetic diversity of the 43 *E*. *ophiuroides* core collections from different geographic locations. A total of 420 alleles were detected in 43 collections, wherein 285 alleles were determined to be collection-specific and 135 alleles were generally detected in multiple collections. The Na amplified per SSR locus ranged from 3 to 13, with an average of 8.40; the Ne ranged from 1.20 to 5.27, with an average of 3.01; the Ho ranged from 0.17 to 0.83, with the average of 0.64. The PIC value ranged from 0.15 to 0.78, with an average of 0.58. The I ranged from 0.31 to 1.76, with an average of 1.17 (Table 4).

**Table 4:** Polymorphism statistics of novel SSR markers in 43 *E*. *ophiuroides* core collections.

ID	Marker name	Na	Ne	Ho	I	PIC
TR1654_c2_g1_i1	TJIB.Eo_004	3	1.88	0.48	0.66	0.36
TR2338_c1_g1_i1	TJIB.Eo_006	10	3.52	0.73	1.41	0.67
TR2941_c0_g1_i2	TJIB.Eo_008	8	5.16	0.83	1.75	0.78
TR4044_c0_g1_i1	TJIB.Eo_009	7	1.40	0.29	0.52	0.26
TR5354_c0_g2_i1	TJIB.Eo_010	11	4.07	0.77	1.65	0.72
TR6729_c0_g1_i1	TJIB.Eo_013	10	2.34	0.59	1.02	0.49
TR7108_c0_g1_i1	TJIB.Eo_014	7	2.59	0.63	1.01	0.54
TR7421_c0_g1_i1	TJIB.Eo_016	5	1.20	0.17	0.31	0.15
TR8741_c0_g1_i1	TJIB.Eo_017	8	3.82	0.76	1.44	0.69
TR9728_c0_g2_i1	TJIB.Eo_020	8	5.27	0.83	1.71	0.78
TR11518_c0_g1_i1	TJIB.Eo_022	7	2.56	0.62	1.14	0.56
TR12898_c0_g1_i1	TJIB.Eo_023	10	5.24	0.83	1.76	0.78
TR13845_c0_g1_i1	TJIB.Eo_025	6	1.90	0.48	0.83	0.42
TR14777_c0_g1_i1	TJIB.Eo_028	5	1.57	0.38	0.55	0.30
TR15964_c0_g1_i1	TJIB.Eo_029	10	2.48	0.61	1.11	0.55
TR16281_c0_g1_i1	TJIB.Eo_031	12	3.36	0.72	1.40	0.65
TR18313_c0_g1_i1	TJIB.Eo_038	11	3.36	0.72	1.34	0.65
TR21460_c3_g4_i1	TJIB.Eo_040	10	3.30	0.71	1.36	0.65
TR23987_c0_g1_i1	TJIB.Eo_044	9	3.38	0.72	1.31	0.65
TR26876_c2_g2_i9	TJIB.Eo_046	11	3.04	0.69	1.22	0.62
TR27357_c0_g3_i1	TJIB.Eo_047	9	4.31	0.79	1.52	0.73
TR29033_c0_g3_i1	TJIB.Eo_051	7	3.74	0.75	1.41	0.69
TR29155_c1_g3_i1	TJIB.Eo_052	13	3.31	0.71	1.26	0.64
TR29883_c0_g1_i4	TJIB.Eo_054	9	2.25	0.57	1.09	0.51
TR30407_c0_g1_i1	TJIB.Eo_055	11	4.68	0.81	1.65	0.76
TR30845_c0_g1_i1	TJIB.Eo_056	9	3.01	0.68	1.28	0.62
TR33948_c0_g1_i1	TJIB.Eo_060	11	2.40	0.60	0.99	0.49
TR34533_c0_g3_i1	TJIB.Eo_061	10	4.50	0.80	1.60	0.74
TR34630_c0_g1_i3	TJIB.Eo_062	7	2.04	0.52	0.81	0.42
TR35413_c0_g1_i1	TJIB.Eo_063	9	1.60	0.39	0.56	0.31
TR39482_c0_g1_i1	TJIB.Eo_065	8	2.98	0.68	1.25	0.60
TR39754_c0_g1_i1	TJIB.Eo_066	10	3.47	0.73	1.35	0.66
TR42732_c0_g1_i1	TJIB.Eo_068	8	2.14	0.55	0.97	0.48
TR45627_c0_g1_i1	TJIB.Eo_071	10	2.62	0.63	1.03	0.55
TR46377_c0_g1_i1	TJIB.Eo_072	10	2.29	0.58	0.98	0.48
TR46548_c0_g1_i2	TJIB.Eo_073	11	1.94	0.50	0.75	0.39
TR46887_c0_g3_i1	TJIB.Eo_074	9	2.86	0.67	1.21	0.59
TR46947_c0_g1_i1	TJIB.Eo_075	8	2.46	0.61	1.04	0.52
TR47761_c0_g2_i1	TJIB.Eo_077	5	1.91	0.49	0.74	0.38
TR48071_c1_g1_i2	TJIB.Eo_078	8	2.65	0.64	1.13	0.57
TR51210_c0_g2_i1	TJIB.Eo_080	9	2.43	0.60	1.21	0.55
TR51733_c0_g4_i1	TJIB.Eo_082	8	3.72	0.75	1.35	0.68
TR52704_c4_g2_i1	TJIB.Eo_084	5	3.07	0.69	1.23	0.61
TR53516_c0_g1_i1	TJIB.Eo_086	9	4.37	0.79	1.62	0.74
TR53584_c1_g1_i1	TJIB.Eo_087	8	5.01	0.82	1.66	0.77
TR53936_c0_g1_i1	TJIB.Eo_089	11	2.70	0.65	1.15	0.57
TR54047_c0_g1_i1	TJIB.Eo_090	4	3.16	0.70	1.25	0.63
TR54725_c0_g4_i1	TJIB.Eo_091	5	2.62	0.63	1.02	0.54
TR54851_c2_g5_i1	TJIB.Eo_092	6	3.07	0.69	1.17	0.61
TR59407_c0_g1_i1	TJIB.Eo_097	5	1.92	0.49	0.97	0.36
Total		420	–	–	–	–
Mean		8.40	3.01	0.64	1.17	0.58

Na, number of alleles; Ne, number of effective alleles; Ho, observed heterozygosity; I, Shannon’s information index; PIC, polymorphism information content.

**Fig 2:**
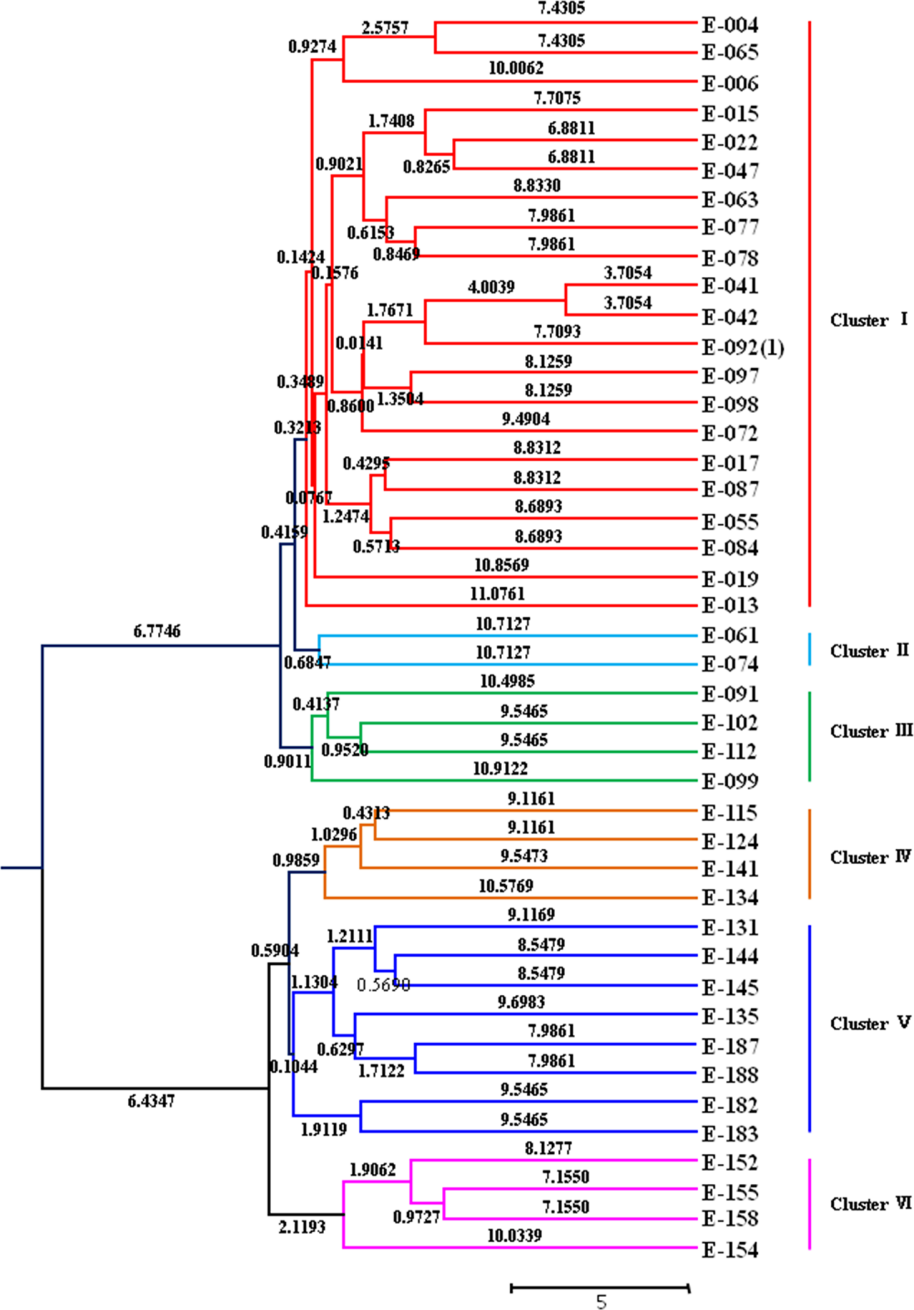
UPGMA-based dendrogram of 43 core collections of *E*. *ophiuroides*.

The genetic distances, with the Nei72 coefficient, among the collections ranged from 0.07 to 0.48 (S6 Table). The largest genetic distance was observed between E188 from Jinzhai, Anhui, Eastern China and E102 from the USA. The UPGMA-based dendrogram placed the 43 accessions into two main groups, which were further subdivided into six distinct clusters (Fig 2). Cluster I comprised 21 collections from Eastern (E004, E006, E047, E042, E017, E019, E013), South (E065, E015, E063, E041, E072, E055), Southwestern (E092-1, E087, E084) and Central China (E022, E077, E078, E097, E098). Cluster II comprised only two accessions from South (E061) and Central China (E074). Cluster III included three accessions from Southwestern (E091, E112) and Eastern China (E099), and one accession from the USA (E102). Cluster IV consisted of four accessions from Central (E115, E124), eastern (E141), and Southwest China (E134). In addition to one collection from Southwestern China (E135), cluster V consisted mainly of the accessions from Central (E131, E144, E145) and Eastern China (E187, E188, E182, E183). Cluster VI contained four collections consisting of one collection from Central China (E152), two from Eastern China (E155, E154) and another one from the USA (E158).

## Discussion

Simple sequence repeats (SSRs) are widely used as molecular markers in plant genetic studies due to their abundance, reproducibility, high allelic variation at each locus and simplicity to analyze using conventional PCR amplification. Recently developed next generation sequencing (NGS) platforms, such as Roche’s 454 GS FLX, Illumina’s GenomeAnalyzer (GA) and ABI’s SOLiD, offer opportunities for high-throughput and inexpensive genome sequencing and rapid marker development. In the present study, SSR markers for the diploid species *E*. *ophiuroides* were developed based on de novo assembled cDNA by the Illumina sequencing system from two phenotypically distinct accessions E092 and E092-1, which were maintained at the China National Germplasm Resources Nursery for warm-season turfgrasses in the Institute of Botany, Jiangsu Province and Chinese Academy of Sciences. The reliability of the newly developed SSR primers was tested via a PCR analysis on the core germplasm collections of centipedegrass, including 41 accessions from China and 2 accessions from the USA.

Centipedegrass is an important warm-season grass species that is widely used for turf in the southeastern United States and in the south of China. Because of its superiorities over other turfgrasses, which mainly include its low maintenance requirements and good adaptation to poor soil, centipedegrass shows great potential for commercial application in the turf industry. However, a lack of genomic information has hampered critical research on augmenting marker assisted breeding programs for this species. Hence, the development of an effective marker system to assess genetic diversity in centipedegrass collections facilitates the maintenance of germplasm and cultivar improvement. Prior to our study, 30 SSR primer pairs were developed in *E*. *ophiuroides* using the traditional method [29]. Recently, approximately 3,500 SSR primer pairs were successfully designed from 5,839 identified SSR loci based on Illumina paired-end sequencing reads [21]. In the present study, a total of 48,061 SSR primer pairs were developed from assembled centipedegrass transcriptome sequences using an NGS-based RNA-seq technique. These SSRs were mined from the transcriptomes, meaning in the gene coding regions only. However, the gene-coding regions should have fewer SSRs than those of the intergenic regions [48]. Therefore, the SSRs developed here are only part of the SSRs in the genome of centipedegrass. Even so, these results represent an important complementation to the molecular toolbox of centipedegrass, which might contribute to promoting genetic and genomic research on centipedegrass.

The motif type and abundance of SSRs are the main characteristics of microsatellites. In this study, the most abundant motif was that of tri-nucleotides (52.14%) followed by di-nucleotides (44.65%), tetra-nucleotides (2.17%), hex-nucleotides (331, 0.51%) and penta-nucleotides (0.49%). This result is consistent with previous findings, showing that tri-nucleotides are the most common type in centipedegrass [21], and is also in accord to the findings in many other plant species, such as alfalfa [49], Dysosma [50], peanut [51], common bean [52], and chrysanthemum [53]. This result indicates that tri-nucleotide repeats are dominant over the other motif types in plant cDNA sequences, which might be explained by the fact that the variations of tri-nucleotide repeats will not affect the open reading frames and only lead to the adding or subtracting of amino acid repeats [54]. AG/CT was the most common motif unit of the grouped SSRs, representing 8.96% of the grouped SSR motif units in the present study, which is common in the genomes of many plant species [49, 55, 56].

In the present investigation, the 100 randomly selected primer pairs were used to check the effectiveness of the actual amplification of all the developed markers. Of these selected primer pairs, 81 (81.0%) of the primers successfully produced amplification in at least one of the four *E*. *ophiuroides* accessions, and 56% successfully amplified alleles from all four centipedegrass accessions, while 19 primers showed no amplification. The unsuccessful amplification indicated that transcriptome-based markers may not work for genomic DNA, which is mainly attributed to the fact that the primer originated from an erroneously assembled transcript or that the primer sequence contained exon-exon junctions. Out of these 81 primers, 50 (61.73%) showed polymorphic patterns, which was lower than the previously reported 87.78% polymorphic genic-SSRs across 14 centipedegrass accessions [21]. In addition to the higher proportion of monomorphic markers, the initial use of a lower quantity of centipedegrass accessions (only four accessions used) for the polymorphism screening was the main reason for the lower proportion of polymorphic markers detected in our study. For the further genetic diversity analysis among the 43 core accessions of *E*. *ophiuroides*, the Na detected by the 50 SSR markers varied from three to thirteen, with an average of 8.40 alleles per locus, while the Ne per locus was from 1.20 to 5.27, with an average of 3.01. This discrepancy can be explained by the fact that quite a few of the alleles identified were collection-common. At the same time, the average Na (8.4) and Ne (3.01) and the Ho and the I detected in the present study were higher than those in a previous study by Wang et al [21], which was because more centipedegrass accessions were used in the present study and because the 43 accessions derived from a core collection of centipedegrass germplasms were preliminarily constructed through a systematic work, including the initial collection of germplasms and the subsequent identification and screening [57]. Moreover, the PIC value is commonly used as a measure of polymorphism for a marker locus and is determined by both the number of alleles and their frequency distribution within the population. According to the classification basis for the marker loci informativeness level proposed by Botstein et al (1980) [58], all the *E*. *ophiuroides* SSRs validated in the present study indicated a moderate level (0.50 > PIC > 0.25) to a high level of informativeness (PIC > 0.5), with an average PIC value of 0.58, with the exception of TJIB.Eo_016 (PIC = 0.15), which was considered a poor marker in these respects. In addition, 70.0% (35 out of 50) of the markers showed a high informativeness level and worked as resolving power markers.

Currently, NGS-based RNA-seq has become one of the most efficient ways for developing genic SSR markers in both model and non-model plants, and many RNA-seq derived SSR markers have been widely utilized in genetic diversity analysis [59,60], chromosome mapping [61,62] and gene-based association studies [63–65]. In the present study, the usefulness of the 50 newly developed polymorphic EST-SSR markers for the evaluation of genetic diversity among centipedegrass accessions was clearly demonstrated. Based on the analysis results, the 50 markers divided the 43 *E*. *ophiuroides* accessions into two main groups and into six subgroups using the UPGMA cluster analysis. Although the resulting dendrogram could not sufficiently cluster the accessions based on their geographical origins, it precisely demonstrated the effectiveness of these SSR markers in the *E*. *ophiuroides* genetic analysis. It is reasonable that the genetic relationship of the 43 *E*. *ophiuroides* accessions did not exactly correspond to their geographical positions due to their complex genetic background and evolution history. Therefore, these large-scale developed SSR markers from centipedegrass transcriptome are valuable tools for further genetic and genomic analyses of centipedegrass accessions. Moreover, Wang et al (2017) [21] reported that centipedegrass EST-SSRs were applicable for six Poaceae relatives and that a higher cross-species transferability of the SSRs was detected in C4 plant maize, sorghum, and sultan grass than that in C3 plant wheat, rice, and barley. A good proportion of the centipedegrass genic SSRs mined in this study also functioned in two other Poaceae C4 members, zoysiagrass and bermudagrass (S1 Fig). Both findings proved the cross-species transferability of the centipedegrass SSR markers to other genetically closed species and reflected the close relationship of the C4 plants. Furthermore, the identification of the EST-SSR within the sequences provides a future opportunity to mine the expressed sequences for significant physical and functional association with turf traits of interest in marker-assisted breeding in *E*. *ophiuroides* and other closely related turf species.

## Conclusions

The present work represents a substantial advance in the identification of a large number of informative SSR loci in *E*. *ophiuroides* by high-throughput RNA sequencing technology based on the Illumina HiSeq 2000 platform. A total of 64,470 SSR loci were identified from the assembled transcriptome of *E*. *ophiuroides*. Among them, the trinucleotide SSRs were the most dominant repeat motif (52.14%), followed by dinucleotides (28,783, 44.65%), tetranucleotides (1,399, 2.17%), hexnucleotides (331, 0.51%) and pentanucleotides (317, 0.49%). In total, 48,061 primer pairs were successfully designed from these identified SSR loci, and a subset of the 100 primer pairs was randomly selected and preliminarily tested in two different types of centipedegrass accessions. The PCR analysis revealed that 81.00% of the primer pairs successfully worked in at least one of the four *E*. *ophiuroides* accessions, and that 56% of the primer pairs successfully amplified alleles from all four accessions. Among the primers tested, 50 (61.73%) of the primers generated polymorphic bands and were further applied to assess the genetic diversity level of 43 centipedegrass core collections. In total, 420 alleles were detected by these newly developed SSR markers in the 43 collections, of which 285 were collection-specific alleles and 135 were multi-accession alleles. The PIC values ranged from 0.15 to 0.78, with an average of 0.58, and the I ranged from 0.31 to 1.76 with an average of 1.17. The 43 *E*. *ophiuroides* core collections were successfully clustered into two main groups and six subgroups based on the UPGMA cluster analysis. Thus, in the present study, the newly developed polymorphic SSR markers successfully shed light on the levels of the molecular diversity inherited in the core collections of centipedegrass deposited in the Turfgrass Research Center of Institute of Botany, Jiangsu Province and Chinese Academy of Sciences (JIB). The SSR primers and sequence information developed in the present study will be useful and robust resources for future genetic and genomic studies, such as genetic map construction and comparative genomic analyses, and molecular marker-assisted breeding in centipedegrass and its related species.

## Supporting information

S1 Fig Examples of PCR amplification of centipedegrass SSR markers in bermudagrass (*Cynodon dactylon* L). M: DNA ladder marker; C1: *Cynodon* accession 118M; C2: *Cynodon* accession 118. The captital letter A, B,…,N represents SSR marker, respectively.
(PPTX)

S1 Table Statistics of *E*. *ophiuroides* SSR mining. (XLSX)

S2 Table Statistics of SSR motif (mer) types of *E*. *ophiuroides*. (XLSX)

S3 Table Statistics of grouped SSR motif units of *E*. *ophiuroides*. (XLSX)

S4 Table Statistics of SSR length of *E*. *ophiuroides*. (XLSX)

S5 Table SSR markers developed from the de novo transcriptome sequence of *E*. *ophiuroides*. (XLSX)

S6 Table Nei's unbiased measures of genetic distance between *E*. *ophiuroides* core collections. (XLS)
