# Supplementary material for: High-throughput SSR marker development and its application in a centipedegrass (Eremochloa ophiuroides (Munro) Hack.) genetic diversity analysis
Source: PLoS One. 2018 Aug 22;13(8):e0202605. doi: 10.1371/journal.pone.0202605 (PMC6105027; doi:10.1371/journal.pone.0202605)
Supplement: S1 Fig — M: DNA ladder marker; C1: Cynodon accession 118M; C2: Cynodon accession 118. The captital letter A, B,…,N represents SSR marker, respectively. (PPTX) [file pone.0202605.s001.pptx]

## Slide 1
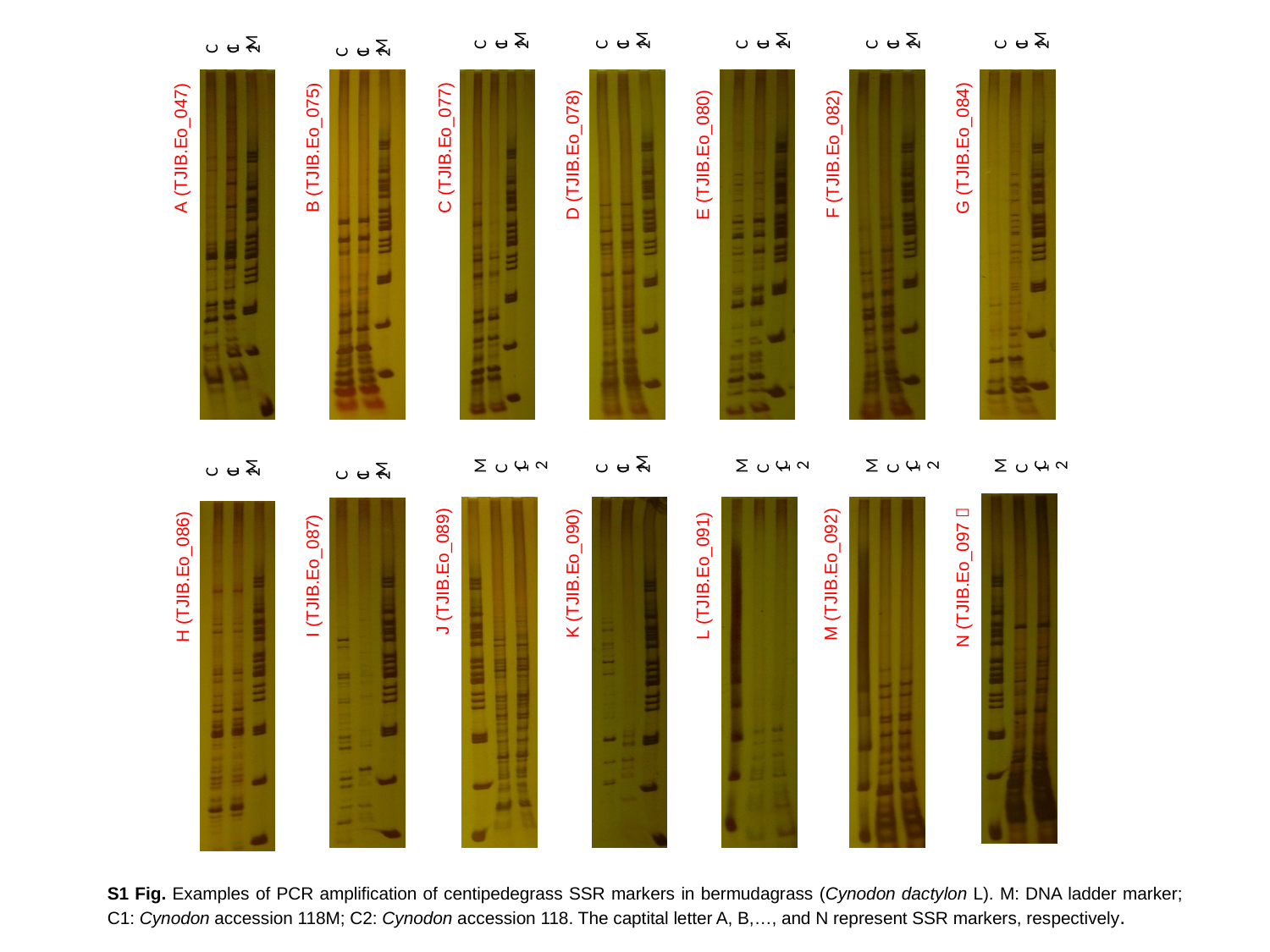

M
C1
C2
M
C1
C2
M
C1
C2
M
C1
C2
M
C1
C2
M
C1
C2
M
C1
C2
G (TJIB.Eo_084)
A (TJIB.Eo_047)
B (TJIB.Eo_075)
C (TJIB.Eo_077)
D (TJIB.Eo_078)
E (TJIB.Eo_080)
F (TJIB.Eo_082)
C2
M
C1
M
C1
C2
C2
M
C1
C2
M
C1
C2
M
C1
M
C1
C2
M
C1
C2
N (TJIB.Eo_097）
J (TJIB.Eo_089)
M (TJIB.Eo_092)
K (TJIB.Eo_090)
L (TJIB.Eo_091)
H (TJIB.Eo_086)
I (TJIB.Eo_087)
S1 Fig. Examples of PCR amplification of centipedegrass SSR markers in bermudagrass (Cynodon dactylon L). M: DNA ladder marker; C1: Cynodon accession 118M; C2: Cynodon accession 118. The captital letter A, B,…, and N represent SSR markers, respectively.
